# Avoiding the echo‐chamber: embracing qualitative research in obstetrics and gynecology to amplify patient voices

**DOI:** 10.1111/aogs.14346

**Published:** 2022-05-05

**Authors:** Taryn Taylor, Andrea N. Simpson, Rohan D’Souza

**Affiliations:** ^1^ Department of Obstetrics and Gynecology Western University London Ontario Canada; ^2^ Center for Education Research & Innovation, Schulich School of Medicine & Dentistry Western University London Ontario Canada; ^3^ Department of Obstetrics and Gynecology University of Toronto Toronto Ontario Canada; ^4^ Li Ka Shing Knowledge Institute St. Michael's Hospital Toronto Ontario Canada; ^5^ Department of Obstetrics and Gynecology St. Michael’s Hospital/Unity Health Toronto Toronto Ontario Canada; ^6^ ICES (formerly the Institute for Clinical Evaluative Sciences) Ontario Canada; ^7^ Department of Obstetrics and Gynecology and Department of Health Research Methods, Evidence and Impact McMaster University Hamilton Ontario Canada; ^8^ Department of Obstetrics and Gynecology and the Lunenfeld Tanenbaum Research Institute, Mount Sinai Hospital University of Toronto Toronto Ontario Canada

One commonly held—yet misguided—assumption about qualitative research is that it makes for a good story but has little merit in informing practice. This is one reason for a relative absence of qualitative research published in clinical obstetrics and gynecology (OBG) journals. While this imbalance in favoring quantitative research is not unique to our specialty, we argue that the unintended consequences of doing so bears particular consideration in OBG. Other related disciplines, such as midwifery, nursing and health professions education have embraced qualitative research as legitimate, impactful and worthy of publication. Qualitative research asks “how?” and “why?” questions in an effort to generate rich knowledge that is contextual and situated.^1^ Thus, we are compelled to ask: “why is qualitative research not well‐established within mainstream clinical OBG journals?” In this editorial, we will discuss common critiques of this approach, highlight the value of rigorous qualitative research within our field and propose a way forward.

In reflecting on reviewer comments and editor responses over the years, we have identified some common critiques that may be driving the apprehension to publish qualitative research. Many qualitative studies are rejected when quantitative metrics of rigor are incorrectly applied to qualitative methodologies. Sampling procedures and sample sizes remain particularly misunderstood. Qualitative research seeks to answer context‐situated questions that may be transferrable, without being generalizable, to other contexts. Sampling is therefore often purposive, driven by the research question and usually shifts based on ongoing analysis. Thus, issues of sampling bias or confirmation bias are largely irrelevant to a qualitative approach. Authors must instead provide rationale for their sampling and final sample size, not based on a power calculation but through the richness of their data, the depth of their analysis and transparency in their sampling process. In this way, qualitative research provides answers to questions that often cannot be answered by using exclusively quantitative approaches.

By centering patient voices, qualitative research can thoughtfully challenge collective biases and blind spots embedded within current guidelines, epidemiological research and clinical trials. For instance, published studies that rely on large datasets suggest a myriad of problematic correlations between self‐reported perinatal cannabis use and adverse fetal and neonatal outcomes.^2^ With these data in hand, clinical practice guidelines understandably conclude that complete abstinence is the only reasonable recommendation.^3^ However, this type of epidemiological research overlooks the motivations and context of pregnant people using perinatal cannabis. In contrast, when qualitative researchers dive into these scholarly conversations, they ask different questions such as: “why do pregnant patients choose to continue cannabis use in pregnancy?”^4^ By conducting semi‐structured interviews, one team found that patients were primarily using cannabis as a form of self‐medication for anxiety, nausea or chronic pain conditions.^4^ Based on their analysis, the authors concluded that the abstinence approach espoused by clinical practice guidelines is likely to stigmatize patients further, leading to non‐disclosure and a missed opportunity to apply a harm‐reduction approach. Guidelines that reflect this patient‐focused research might emphasize the importance of focusing on understanding why a patient may be choosing to use cannabis, perhaps to offer alternative symptom management, rather than simply advising abstinence.

If we continue to overlook the value of qualitative research, and patient voices by proxy, we also risk creating an echo‐chamber that reinforces inaccurate assumptions about what matters most to patients. When designing and conducting prospective studies, most clinicians and researchers rely on a biomedical model of health, which presumes that physical health is more important than mental, social and functional wellbeing. Yet health service users’ decision‐making aligns more with a biopsychosocial model of health which is “a state of complete physical, mental and social well‐being and not merely the absence of disease or infirmity”, as defined by the World Health Organization.^5^ Qualitative research provides a valuable opportunity to understand health from this holistic point of view and explore patient perspectives to ensure that patient‐important outcomes are prioritized. Failure to incorporate these outcomes into clinical studies results in research wastage from studies not being powered to assess outcomes that are relevant to patients. For instance, a meta‐synthesis of qualitative literature regarding chronic pelvic pain revealed that the standard outcomes typically assessed in clinical trials fall perilously short of capturing the full patient experience.^6^ While many clinical trials simply report patient “satisfaction” as an outcome,^7^ this meta‐synthesis reveals a more comprehensive understanding of satisfaction that includes attention to psychosocial impacts of chronic pelvic pain, interactions within the healthcare system and a desire for individualized care.^6^

Clinical guidelines on pregnancy‐related conditions also contain embedded assumptions about preferred trade‐offs between maternal and fetal health. For example, clinical practice guidelines tend to recommend anticoagulant regimens involving vitamin‐K antagonists (VKAs) over low molecular weight heparin (LMWH) for pregnant individuals with mechanical heart valves despite known fetotoxic effects of VKAs, on the basis that VKAs are associated with fewer maternal deaths and thromboembolic complications.^8^ However, a global survey of healthcare professionals showed that after considering absolute numbers and the pros and cons of LMWH vs VKAs, many patients opt for LMWH, despite the higher maternal risks.^9^ This suggests that decision making involves more than the consideration of relative risks; it also involves making trade‐offs based on the nature, severity and absolute risk of maternal and fetal/neonatal outcomes along with the long‐term biopsychosocial implications—issues that cannot be thoroughly explored through quantitative methods alone. Evidence‐based medicine requires that clinical decisions be consistent with the informed values and preferences of the patient. A qualitative study design enables researchers to explore the reasons behind patient decisions, including values and preferences, to inform clinical practice guidelines.

Qualitative research conducted before a randomized controlled trial (RCT) can inform us not only on how best to select outcomes and design the RCT, but also whether conducting the RCT is feasible. For example, a group of researchers designed an RCT to explore whether dalteparin or low dose aspirin (ASA) could effectively reduce the risk of recurrent pregnancy loss in patients with anti‐phospholipid antibody syndrome. Concurrently, the group conducted a qualitative study that explored the perspectives of patients with these conditions.^10^ The study revealed that recruitment for the RCT would be nearly impossible, as the chance of receiving placebo was intolerable to most participants based on the desire to have a successful pregnancy at all costs and despite unproven benefits of treatment. As this example illustrates, research that informs patient‐centered decision making involves more than a simple calculation of odds ratios and relative risks—it also requires that we account for patient values and perspectives.

Qualitative research methodologies are also well‐suited to grapple with nuance, complexity and the “wicked problems” of real life. Interprofessional conflict, as one example, is both unwieldy and a major threat to patient safety. In their study of interprofessional tensions on a labor and delivery unit, Brydges et al. used a qualitative methodology called Institutional Ethnography to expose specific work processes, policies and guiding texts (eg remuneration structures) that were unintentionally compromising patient safety.^11^ Their findings have informed concrete changes within the study context that are potentially transferable to other labor and delivery units. This study exemplifies the exploratory nature of qualitative research while demonstrating that hypothesis‐generating research can be tremendously impactful.

The above examples highlight how qualitative research has tremendous potential to shape healthcare policies and practices that are truly patient‐centered. The vast range of available methodologies provides the flexibility to address diverse and salient research questions, as we have highlighted in the examples above. Exposing epidemiologists, clinicians and clinical researchers to qualitative and mixed methods research during training will better equip them to interpret and critically appraise qualitative work. Quantitative researchers would do well to partner with qualitative experts and patient representatives during the planning of large‐scale trials to ensure the final research product matters. Mainstream journals could improve existing processes for reviewing and adjudicating qualitative research submissions, which is achievable by increasing the number of reviewers with familiarity in qualitative methodologies and ensuring that editorial boards include members with the required expertise. Journals may also consider creating a subsection for qualitative research methods in each issue. Of course, qualitative scholars also have an ongoing responsibility to submit original research that is accessible, compelling and relevant to those unfamiliar with qualitative research. Although the black‐and‐white world of quantitative research is familiar and well‐entrenched, for the benefit of our patients and the future of our profession, it is time to embrace qualitative research in OBG.
